# Comparison of Microbial Populations in the Blood of Patients With Myocardial Infarction and Healthy Individuals

**DOI:** 10.3389/fmicb.2022.845038

**Published:** 2022-05-25

**Authors:** Ikram Khan, Imran Khan, Mian Adnan Kakakhel, Zhang Xiaowei, Mao Ting, Ikram Ali, Yu Fei, Zhou Jianye, Li Zhiqiang, An Lizhe

**Affiliations:** ^1^School of Life Sciences, Lanzhou University, Lanzhou, China; ^2^School of Stomatology, Northwest Minzu University, Lanzhou, China; ^3^Department of Microbiology, Khyber Medical University Peshawar, Peshawar, Pakistan; ^4^Lanzhou University Second Hospital, Lanzhou, China

**Keywords:** blood circulation, bacteria, functional analysis, myocardial infarction, HiSeq

## Abstract

Increased bacterial translocation in the gut and bloodstream infections are both major comorbidities of heart failure and myocardial infarction (MI). However, the alterations in the microbiome of the blood of patients with MI remain unclear. To test this hypothesis, we conducted this case-control study to explore the microbiota compositions in the blood of Chinese patients with MI. Using high-throughput Illumina HiSeq sequencing targeting the V3–V4 region of the 16S ribosomal RNA (rRNA) gene, the microbiota communities in the blood of 29 patients with MI and 29 healthy controls were examined. In addition, the relationship between the blood microbiome and clinical features of MI was investigated. This study revealed a significant reduction in alpha diversity (Shannon index) in the MI group compared with the healthy controls. Also, a significant difference was detected in the structure and richness between the patients with MI and healthy controls. The members of the phylum Actinobacteria, class Actinobacteria, order Bifdobacteriales, family Bifidobacteriaceae, and genus *Bifidobacterium* were significantly abundant in the MI group, while the members of the phylum Bacteroidetes, class Bacteroidia, and order Bacteroidales were significantly enriched in the healthy controls (*p* < 0.05). Moreover, the functional analysis revealed a significant variation between both groups. For instance, the enrichment of genes involved in the metabolism pathways of three amino acids decreased, that is, nucleotide transport and metabolism, coenzyme transport and metabolism, and lipid transport and metabolism, among others. Our study will contribute to a better knowledge of the microbiota of blood, which will further lead to improved MI diagnosis and therapy. Further study is needed to determine the role of the blood microbiota in human health and disease.

## Introduction

Cardiovascular diseases (CVDs), particularly myocardial infarction (MI), remain a major cause of mortality and morbidity around the world (Basha et al., 2020; Li et al., 2021). Every four out of five CVD deaths are caused by stroke or MI (Gomes and Paiva, 2021; Tesfaye et al., 2021). Although treatment procedures like percutaneous coronary intervention (PCI) have reduced the acute MI mortality rates, a growing body of evidence indicates that the incidence of cardiovascular events following an MI still predicts a higher mortality risk (Zhou et al., 2018). MI is a complex disease that is influenced by a variety of factors. Among them, the influence of factors, such as lifestyle, genetics, environment, and gut microbiota, has been largely investigated (Lourenςo et al., 2018; Zununi Vahed et al., 2018). However, the role of blood microbiota composition in MI is not well-investigated.

Several studies reported that bacteria and bacterial metabolites play a systemic role in the development of CVDs (Ahmadmehrabi and Tang, 2017; Amar et al., 2019). For instance, two decades ago, a population-based study revealed an association between endotoxemia (an increase in the endotoxin levels produced by Gram-negative bacteria in the blood) and atherosclerosis (Wiedermann et al., 1999). A bacterial metabolite, trimethylamine oxide, has recently been demonstrated to have a detrimental effect on the walls of blood vessels in an animal model (Chen et al., 2017), and the significance of this discovery in humans has been highlighted in a large prospective population-based investigation (Tang et al., 2013). Thanks to the recent advances in the new discipline of human metagenomics, changes in the gut microbiota have been discovered in individuals with symptomatic atherosclerotic disease (Nielsen et al., 2012). In addition, a diverse microbiome has been observed within the human atherosclerotic plaques (Nielsen et al., 2012; Jonsson et al., 2017), suggesting a role for bacterial translocation in the development of atherosclerosis. Several MI-related changes could result in bacterial translocation and alterations in the blood microbiome communities. In particular, increased translocation of intestinal bacteria (“leaky gut”) and bloodstream infections are found to be typical comorbidities associated with heart failure and MI, and the role of bacteria in the onset of CVDs has also been established (Krack et al., 2005; Zabell and Tang, 2017). As a result, significant hemodynamic changes associated with post-MI and ventricular dysfunction, leading to intestinal hypoperfusion and congestion, have been shown to alter gut functions, morphology, and permeability, thus affecting gut microbiota composition and increasing bacterial translocation to the bloodstream (Sandek et al., 2007, 2014). Indeed, recent research has proved and analyzed the influential role of gut microbiota in innate immunity and its profound impact on the complications related to CVDs (Wang et al., 2011; van den Munckhof et al., 2018). Simultaneously, the role of oral microbiota in the etiology of these diseases has been extensively investigated. Studies on blood microbiota provide the first line of evidence for the involvement of tissue microbiota in CVD, as well as provide some insight into the mechanism of action of the circulating bacteria (Dinakaran, 2017).

The microbiota plays a crucial role in the immunity and inflammation of the host immune system, particularly in the systemic circulation (Yarur et al., 2011). Blood in healthy organisms is considered to be a sterile environment, owing to the lack of proliferating microbes (Potgieter et al., 2015). However, the existence of completely sterile blood in healthy humans has been challenged. (Nikkari et al., 2001) found that even “healthy” blood samples can contain bacterial 16S rDNA. Recently, viable microbes have been discovered in the blood of patients suffering from certain non-communicable diseases, such as type 2 diabetes (Amar et al., 2011a), CVD (Amar et al., 2013), liver fibrosis (Lelouvier et al., 2016), and chronic kidney disease (Shah et al., 2019). This raises the question about the functions of blood microbiota and their impact on MI. Furthermore, Lehtiniemi et al. (2005) identified known members of the oral microbiota in coronary artery tissues, indicating that the bacteria had translocated from the oral cavities into the bloodstream, possibly as a result of damage caused by toothbrushing or leaking across the mucosal surfaces. However, it is still unclear whether a complex bacterial community exists in the blood of patients with MI. Therefore, it is crucial to investigate the bacterial profile of blood in patients with MI, to better understand their association with MI which might be helpful as a module for future treatment plans.

Based on previous studies, we hypothesized possible alterations in the microbiota of the blood of patients with MI. This study aimed to characterize the blood microbial composition of MI patients and to compare them with healthy controls, using 16S rRNA gene sequencing. The major goal of analyzing the blood bacterial community was to determine whether a particular blood microbiota population is associated with a disease-specific condition. We also analyzed the relationships between the microbiota present in the blood of patients with MI and the clinical characteristics of MI. Variation in the blood microbiome community will provide crucial information on the genesis, causes, and effects of inflammation in the MI population.

## Materials and Methods

### Recruitment of Patients and Volunteers

The study was approved by the Ethics Committee of Northwest Minzu University in Lanzhou, Gansu, China. All subjects provided written informed consent following the Declaration of Helsinki.

A total of 48 volunteers were recruited for this study, which included 29 patients with MI (23 men and 6 women) and an equal number of men and women were selected for the healthy group (HC). The patients diagnosed with MI were recruited in the cardiology ward, while healthy participants were selected at the physical examination center of Lanzhou University Second Hospital, China. Exclusion criteria for both the groups were as follows: development of infectious disease within 1 week prior to inclusion, immunocompromised individuals, taking antibiotic treatment within 1 month before the inclusion, pregnancy, and suffering from chronic viral infections [i.e., hepatitis C, human immunodeficiency virus (HIV), and herpes simplex type 2], chronic inflammatory intestinal bowel disease, and renal failure. All the study subjects had a history of MI. A majority (25/29; 86%) of patients were included within 10 days of an acute ischemic event. Moreover, demographic, clinical, and biological data of each participant were recorded, and participants with missing data were excluded from the study.

### Collection of Blood Samples

Clinically certified team members collected venous blood samples in Vacutainer EDTA Blood Collection Tubes. Reagents and materials were disinfected, and medical staff wore lab clothes, masks, and disposable gloves to avoid contamination by foreign DNA. For each volunteer, 3 ml of blood sample was drawn in the morning after following overnight fasting conditions and stored at −80°C for further use.

### DNA Extraction and Sequencing

Total bacterial DNA was extracted from 200 μl of whole blood (in EDTA) according to the manufacturer's procedure using Power Soil DNA Isolation Kit (MO BIO Laboratories). NanoDrop was used to test the quality and quantity of DNA using the 260/280 nm and 260/230 nm ratios (Biomarker Technologies Corporation, Beijing, China). Furthermore, the DNA was preserved at −80°C until further processing.

According to Illumina guidelines, a library for 16S metagenomic sequencing was generated. Metagenomic sequencing was used to create libraries and amplify the bacterial 16S rRNA gene. Forward primer 341 (5′-ACTCCTACGGGAGGCAGCA-3′) and reverse primer R806 (5′ - GGACTACHVGGGTWTCTAAT-3′) were combined with adapter and barcode sequences to amplify the hypervariable region V3–V4 of the bacterial 16S rRNA gene. A total volume of 50 μl was used for PCR amplification, which included 10 μl of buffer, 0.2 μl of Q5 high-fidelity DNA polymerase, 10 μl of high GC enhancer, 1 μl of dNTPs, 10 μM of each primer, and 60 ng of genomic DNA. The thermal cycling conditions were as follows: initial denaturation at 95°C for 5 min, then 20 cycles at 95°C for 1 min, 50°C for 1 min, and 72°C for 1 min, followed by a final extension at 72°C for 7 min. VAHTSTM DNA Clean Beads were used to purify the PCR products from the first-phase PCR. A 40-μl reaction mixture containing 20 μl of 2 × Phμsion HF MM, 8 μl of ddH_2_O, 10 μM of each primer, and 10 μl of PCR products obtained from the first step was used to perform the second phase of PCR experiments. The thermal cycling conditions were as follows: initial denaturation at 98°C for the 30 s, then 10 cycles at 98°C for 10 s, 65°C for 30 s, and 72°C for 30 s, with a final extension at 72°C for 5 min. Finally, the Quant-iT^TM^ dsDNA HS reagent was used to quantify all the PCR products, which were then pooled together. High-throughput sequencing analysis was performed on purified bacterial 16S rRNA genes, and Illumina HiSeq 2500 (2 × 250 paired ends) platform was used to analyze a pooled sample at Biomarker Technologies Corporation, Beijing, China. Sample of sterile water was used as a negative control to purify of DNA libraries (Supplementary Figure 1).

### Bioinformatics and Statistical Analysis

To perform quality filtering on the original data, Trimmomatic version 0.33 was used (Bolger et al., 2014). Cutadapt (version 1.9.1) was used for the further identification and removal of primer sequences (Martin, 2011), FLASH version (1.2.11) was used to remove terminal reads (Magoč and Salzberg, 2011), and UCHIME (version 8.1) was used for removing chimera (Edgar et al., 2011), and finally, a high-quality sequence was obtained for subsequent analysis. Next, sequences smaller than 100 bp and an error rate >2 were eliminated using USEARCH (version 10.0). The Database Project of Ribosomes was used to classify the representative sequences (Cole et al., 2009). More than 97 percent of identical clusters were added to operational taxonomic units after data quality was improved. R studio (version 3.2.1) was used to perform VENN analysis and construct rarefaction curves at the OTU level (Colwell et al., 2012). QIIME2 software (https://qiime2.org/) was used to analyze the alpha index, which included the Chao1 index, Shannon–Wiener diversity index, Simpson diversity index, phylogenetic diversity (PD) index, abundance-based coverage estimator (ACE), and coverage. The R language platform (version 3.2.1) was used to perform principal coordinate analysis (PCoA) based on the Bray-Curtis distance matrix to measure beta diversity. Community variance was examined by permutational multivariate analysis of variance (PERMANOVA) with the Bray-Curtis similarity index. Microorganism features used to distinguish the blood microbial specific to MI were identified using Linear discriminate analysis LDA effect size LEfSe method (Segata et al., 2011). Linear discriminate analysis (LDA), combined with standard tests for the measurement of significant differences (Kruskal–Wallis test and pairwise Wilcoxon test), provides better information regarding the relationship between different species. Phylogenetic investigation of communities by reconstruction of unobserved states (PICRUSt2) was used to predict the functions of blood microbial communities of both groups.

Descriptive data were expressed as mean values and percentages for continuous variables and frequencies for categorical variables. The important features of both the groups were first compared using Fisher's exact test to determine the non-random association between the categorical variables (including being male, hypertension, diabetes mellitus, and current smoking status) of both groups. The Mann–Whitney test was used to compare the differences in quantitative variables (including age, BMI, systolic blood pressure, diastolic blood pressure, TG, LDL, and HDL) between both the groups. Benjamini–Hochberg method was used to control the false discovery rate (FDR) and minimize type 1 error. Spearman's rank correlation test was performed between clinical parameters and the reported phyla. Also, Wilcoxon signed-rank test was used to compare the relative abundance for the difference in the function based on the COG pathway. All the statistical analyses of clinical data were conducted using Prism version 8. The significance was judged by a *p* < 0.05.

## Results

### Clinical Characteristics of HC and MI

Baseline characteristics of the patients and controls are given in Table 1. The patients with MI were older than HC, and no significant differences in gender were found between patients and HC. However, the smoking ratio was significantly higher in the patients with MI (*p* < 0.05) (Supplementary Table 1). In addition, no significant differences were observed in the quantitative variables, such as body mass index (BMI), triglyceride levels (TG), hypertension, diabetes mellitus (DM), systolic blood pressure, diastolic blood pressure, low-density lipoprotein (LDL), and high-density lipoprotein (HDL), between the two groups (Supplementary Table 2). As we performed multiple comparisons, we used the Benjamini–Hochberg method to control the FDR with an acceptable level of *p* < 0.05 to minimize Type I error. When the individual *p*-value was less than the Benjamini–Hochberg value, it indicated that the variable was significant.

**Table 1:** Participant characteristics.

**Variable Mean ±SD or *n* (%)**	**MI patients**	**Healthy individuals**	***P***
Age	59.24 ± 12.90	56.51 ± 12.47	1.13
BMI	24.94 ± 4.02	24.96 ± 4.00	1.06
Sex (male)	23 (79.3%)	23 (79.3%)	0.99
Hypertension	9 (31.03%)	11 (37.9%)	1.07
Diabetes mellitus	4 (13.7%)	6 (20.6%)	1.33
Current smoking	15 (51.72%)	1 (3.4)	0.001**
Systolic BP (mmHg)	123 ± 26	118 ± 17	1.08
Diastolic BP (mmHg)	71.55 ± 16.25	75 ± 14	0.80
TG, mmol/L	1.5 ± 0.84	1.68 ± 1.26	1.20
LDL mmol/L	2.7 ± 1.5	2.5 ± 0.7	1.00
HDL mmol/L	1.06 ± 0.25	1.16 ± 0.20	0.47

*Statistically significant p-values after applying the Benjamini–Hochberg procedure with a false discovery rate of p <0.05*.

BMI, body mass index; TG, triglyceride; LDL, low-density lipoprotein; HDL, high-density lipoprotein. (**shows strong significant results).

### Distribution of OTUs

**Figure 1:**
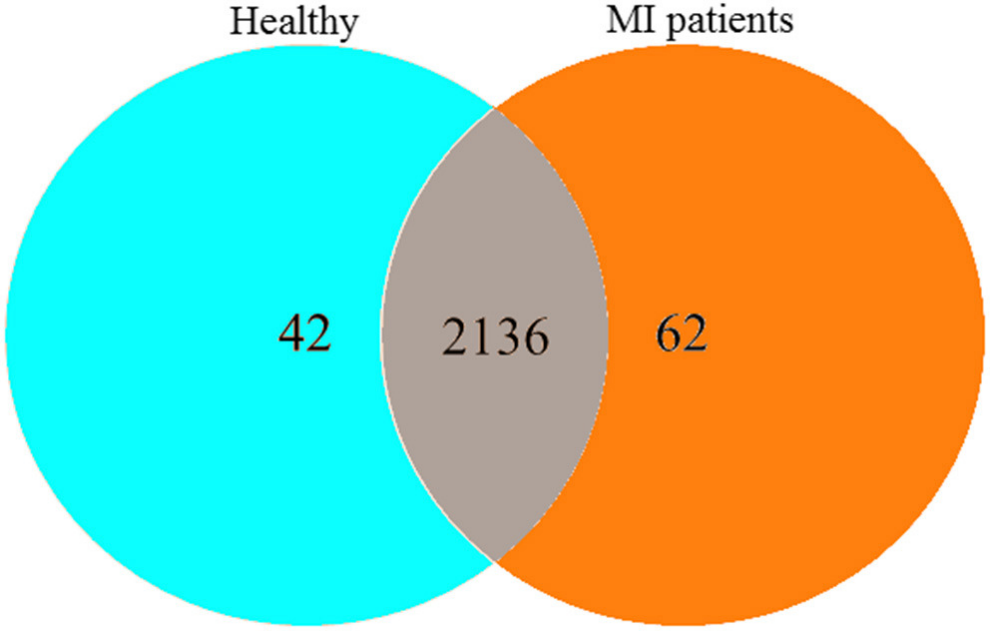
The Venn diagram represents the shared and unique OTUs in both MI and HC groups.

After sequencing 58 blood samples, a total of 4,408,101 pairs of reads were obtained. Then, to improve the quality, splicing of the double-ended reads was done, and 4,338,707 clean reads were generated. Each sample generated at least 44,209 clean reads, with an average of 74,805 clean reads. Finally, a total of 2,240 OTUs were obtained (Figure 1).

### Alpha Diversity of Blood Bacterial Community

The alpha diversity indices for the MI group and controls are given in Table 2. To account for the differences in diversity and richness distribution of blood bacterial community between HC and MI groups, several indices, such as ACE, coverage, Shannon (diversity), Simpson (diversity), Chao1 (richness), and PD, were estimated. Shannon diversity index was found to be significantly lower in the MI group when compared to the control group (8 ± 0.3 vs. 7 ± 01; *p* = 0.0199), while ACE and Chao1 and PD indices were found to be significantly higher in the MI group when compared to the healthy group.

**Table 2:** Alpha diversity indices between both groups.

**Alpha diversity**	**Healthy control**	**MI patients**	***p***
ACE index	1566.6001 ± 362	1777.8631 ± 217	0.0093**
Chao1 index	1215.2772 ± 223	1319.0905 ± 106	0.028*
PD index	81.3174 ± 20	97.387 ± 12.06	0.0006**
Shannon index	8.0363 ± 0.3	7.4868 ± 1.19	0.0199*
Simpson index	0.9899 ± 0.003	0.965 ± 0.12	0.2866

Alpha diversity showed significant differences between both groups that are denoted with (1*) and (2**). (*) indicates significance and (**) shows strong significance. ACE, abundance-based coverage estimators; PD, phylogenetic diversity.

### Structure of Blood Bacterial Community

**Figure 2:**
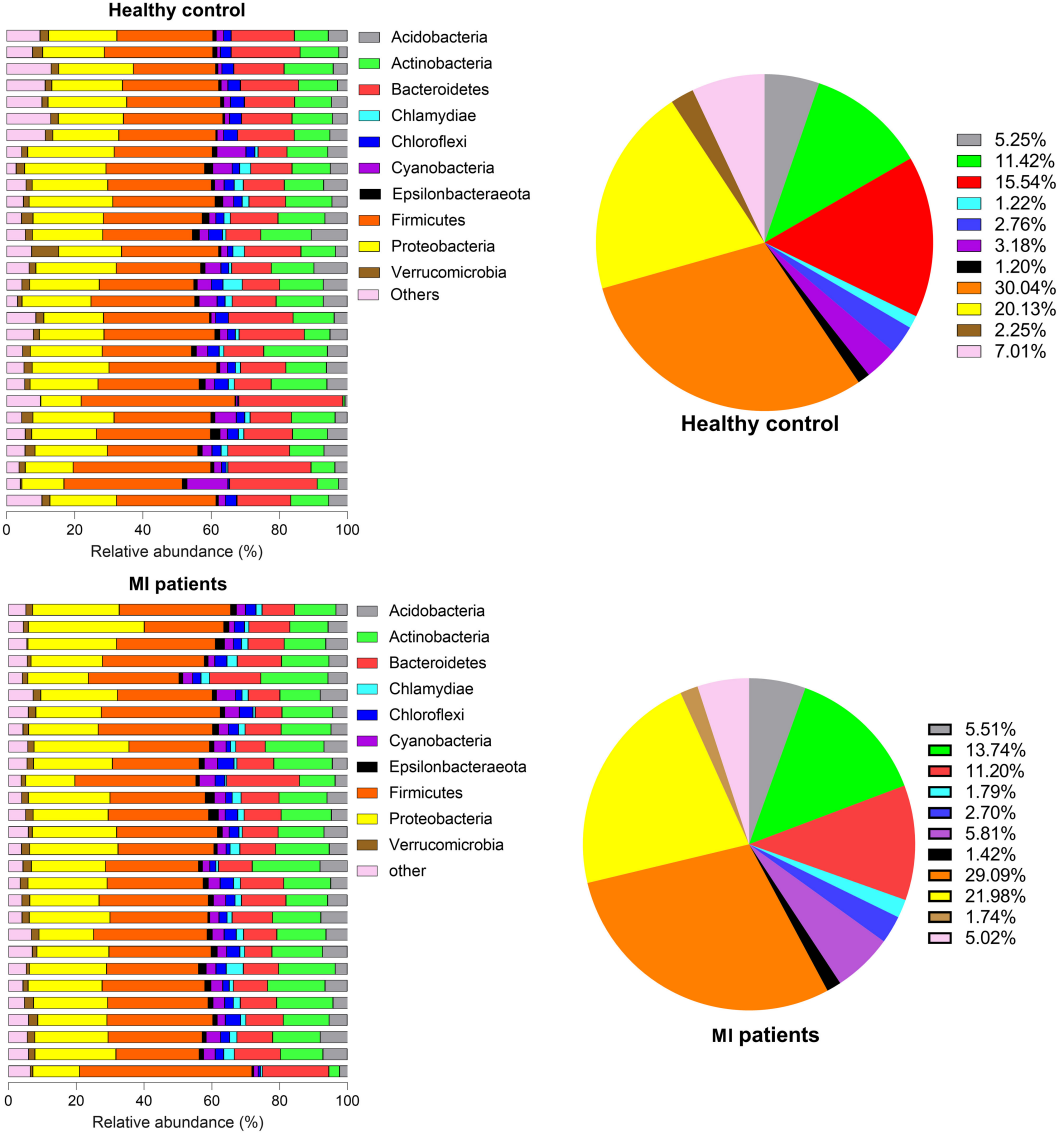
Phylum proportion of HC and MI groups. Pie charts indicate the percentages of each of the top 10 high-abundance phyla in both groups.

The relative abundance of the top 10 OTUs was classified at phylum and genus levels. At the phylum level, the major phyla were Firmicutes and Proteobacteria, together accounting for nearly 90% of the total sequences. Other phyla were Bacteroidetes, Actinobacteria, Acidobacteria, Cyanobacteria, Chloroflexi, Verrucomicrobia, Chlamydiae, and Epsilonbacteria. When the occurrence of these phyla was compared between HC and MI groups, a noticeable increase in the members of phyla Firmicutes (3 ± 7.98 vs. 2.91 ± 1.3; *p* = 5.60), Bacteroidetes (1.5 ± 9.3 vs. 1.2 ± 6.6; *p* = 9.99), Chloroflexi (2.77 ± 1.8 vs. 2.70 ± 2.03; *p* = 9.40), and Verrucomicrobia (2.26 ± 2.42 vs. 1.75 ± 1.24; *p* = 6.59) was observed in the HC group, whereas Proteobacteria (2.01 ± 6.04 vs. 2.20 ± 7.87; *p* = 8.39) followed by Actinobacteria (1.14 ± 6.13 vs. 1.37 ± 7.45; *p* = 9.49), Acidobacteria (5.25 ± 3.83 vs. 5.52 ± 3.36; *p* = 6.14), Cyanobacteria (3.19 ± 4.53 vs. 5.81 ± 2.72; *p* = 5.43), Chlamydiae (1.22 ± 2.48 vs. 1.79 ± 1.84; *p* = 7.09), and Epsilonbacteria (1.20 ± 1.16 vs. 1.42 ± 1.16; *p* = 1.94) were found in high abundance in the MI group (Figure 2).

**Figure 3:**
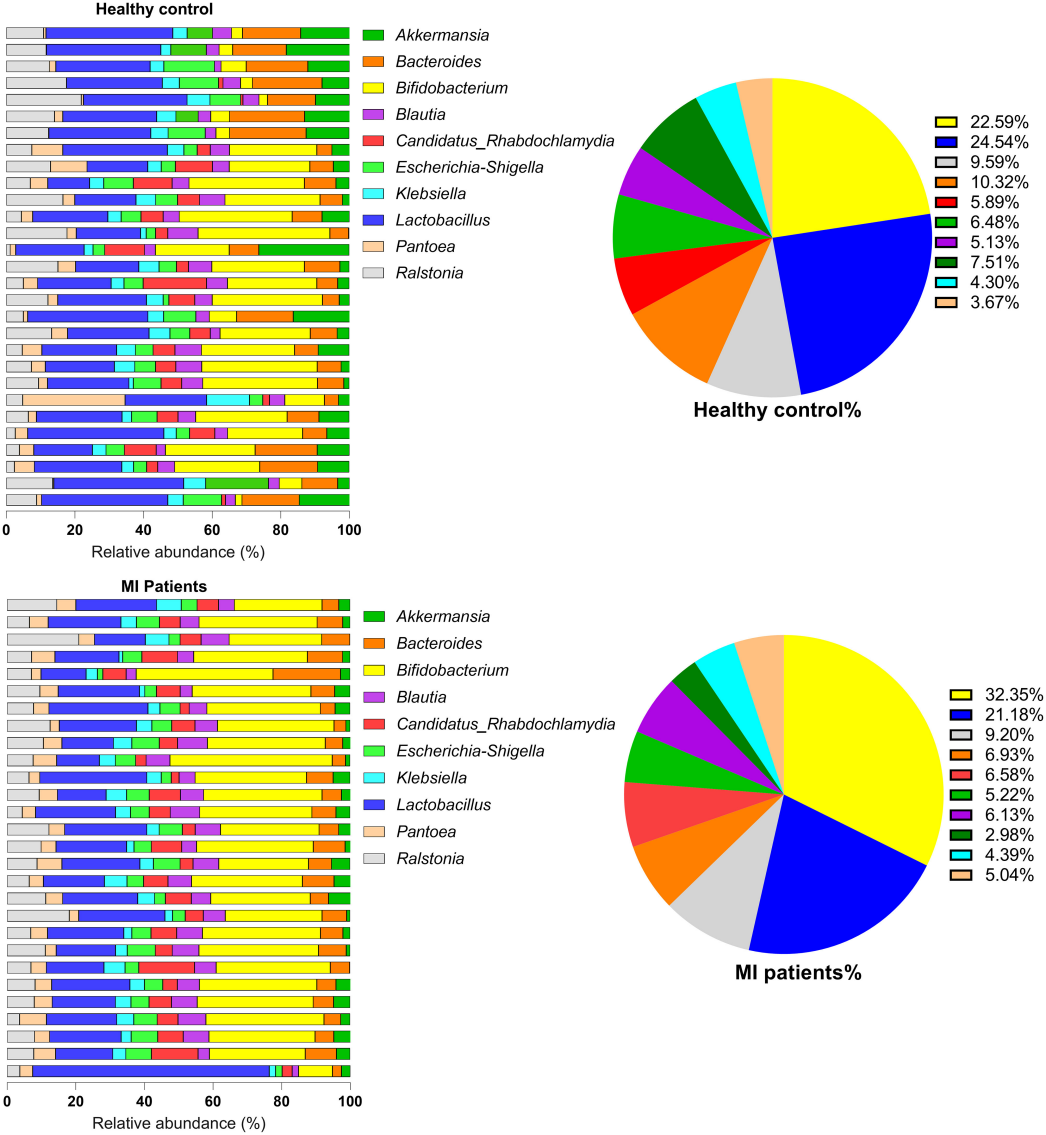
The genus-level abundance of HC and MI groups. Pie charts indicate the percentages of each of the top 10 high-abundance genera in both groups.

At the genus level, the most dominant genera were *Bifidobacterium, Lactobacillus, Ralstonia, Bacteroides, Candidatus Rhabdochlamydia, Escherichia/Shigella, Blautia, Akkermansia, Klebsiella*, and *Pantoea*. Of these, *Bacteroides* species (2.05 ± 1.35 vs. 1.9 ± 1.31; *p* = 6.33) were found in a higher proportion in the HC group. However, *Bifidobacterium* (4.64 ± 6.62 vs. 8.70 ± 5.25; *p* = 9.99) followed by *Lactobacillus* (4.9 ± 3.0 vs. 5.74 ± 3.90; *p* = 8.09), *Ralstonia* (1.92 ± 2.44 vs. 2.48 ± 2.41; *p* = 1.22), *Escherichia/Shigella, Candidatus, Rhabdochlamydia, Klebsiella, Blautia*, and *Pantoea* species were found in increased proportion in the MI group (Figure 3). The relative abundance of bacterial phyla and genera was analyzed using metastatic samples. No significant differences were observed between both the groups at genus and phylum levels (*p* ≤ 0.05).

### Beta Diversity of Blood Bacterial Community

**Figure 4:**
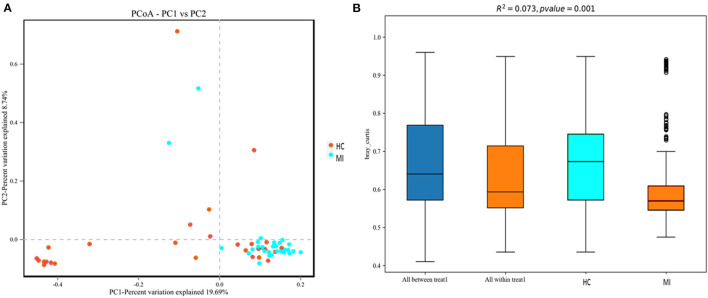
**(A)** Principal coordinate analysis (PCoA) of the overall composition of the genera communities among both groups. **(B)** PERMANOVA analysis indicates variation in blood bacterial species between HC and MI groups.

To investigate structural differences in blood bacterial community between both groups, we assessed the beta diversity index. The overall differences were visualized using a principal coordinate analysis (PCoA) plot. The diversity described in the PCoA plot by the top two principal coordinates was as follows: PC1 = 19.69% and PC2 = 8.74%. A total of 28.43% variation was observed based on the Bray–Curtis distance, as depicted in Figure 4A. In addition, according to PERMANOVA results, the microbial composition between MI patients and HC was significantly different (*R*^2^ = 0.073), (*p* = 0.001) (Figure 4B). Moreover, the hierarchical clustering tree based on the Bray–Curtis analysis revealed that the blood bacterial community showed dissimilarities between the patient and control groups (Supplementary Figure 2). A moderate separation in beta diversity between both groups was observed from principal coordinate analysis based on Bray–Curtis distance.

### Differential Analysis

**Figure 5:**
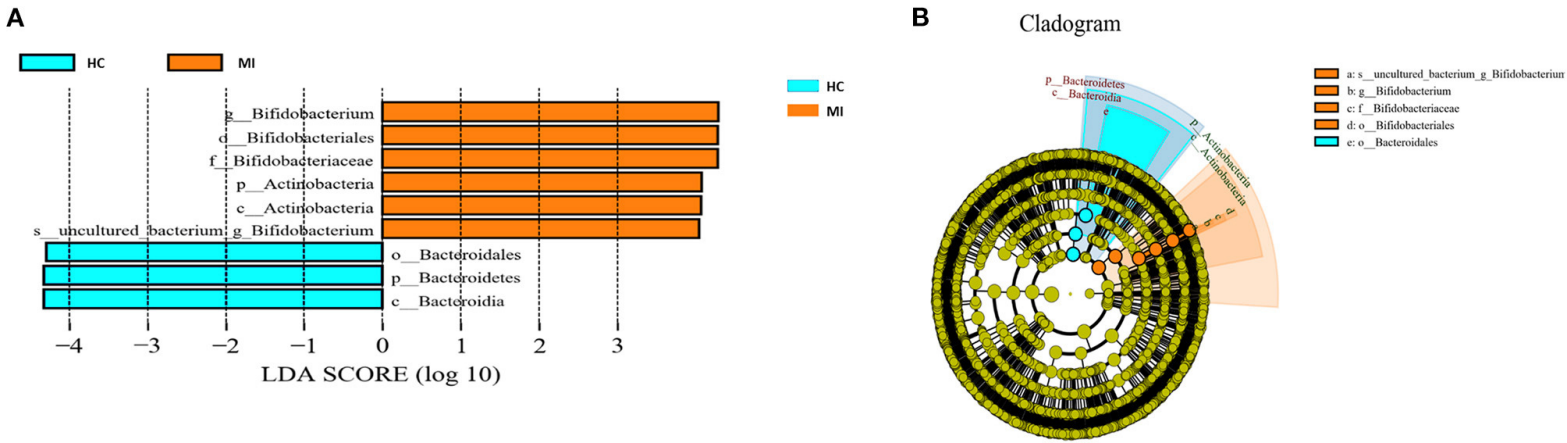
**(A)** LEfSe analysis plot of differentially abundant blood microbial taxa between MI and control. **(B)** The taxonomic tree of differentially abundant taxa is represented by the cladogram.

Different taxonomy levels were compared using LEfSe analysis based on the non-parametric factorial Kruskal–Wallis rank-sum test to identify bacterial taxa with significant differential abundance between the MI group and HC group, to categorize the specific microbial taxa linked with MI (Figure 5A). LEfSe was used to estimate the biomarkers (effect size) of each differentially abundant taxa with the criteria of LDA ≥ 4.0 and *p* < 0.05. A total of nine taxa were differentially abundant in the MI group and the control group. Among these taxa, phylum Bacteroidetes, class Bacteroidia, and order Bacteroidales were highly enriched in HC, while phylum Actinobacteria, class Actinobacteria, order Bifidobacteriales, family Bifidobacteriaceae, and *Bifidobacterium* genus were significantly higher in MI group. The cladogram shows the phylogenetic distribution of blood microbes associated with MI patients using the LEfSe method, where orange color indicates phylotypes statistically over-represented in MI patients and blue color indicates phylotypes over-represented in HC. Each phylotype is represented by a filled circle in the cladogram, and the order, families, genus, and species are listed in the right panel (Figure 5B).

### Correlation Between Clinical Parameters and MI Taxa

**Figure 6:**
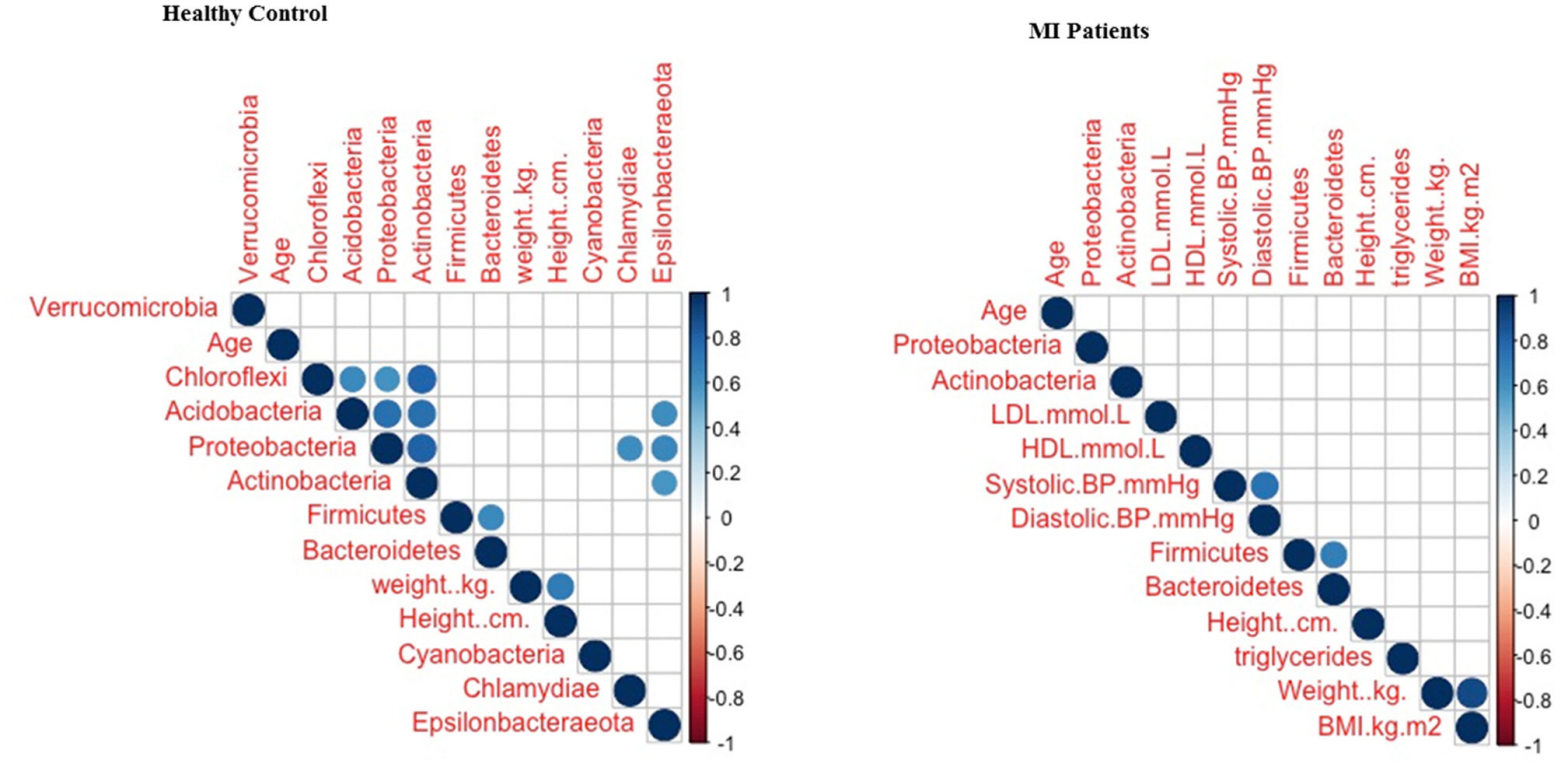
The correlation between clinical parameters and blood bacterial taxa.

The correlation between clinical parameters and MI taxa based on phylum level was used to assess the influence of the clinical parameters on blood bacterial structure and composition. The current findings revealed that there was no significant influence of clinical parameters on bacterial diversity (Figure 6). Hence, it was concluded that MI might have a significant impact on the alteration of the blood bacterial composition.

### Functional COG Pathway

To further investigate the biological and functional properties of all differentially expressed proteins, the present study used COG annotation. In this study, a total of 24 functional categories were predicted. The results demonstrated that these 14 COG functions, including RNA processing and modification, chromatin structure and dynamics, energy production and conversion, amino acid transport and metabolism, carbohydrate transport and metabolism, coenzyme transport and metabolism, cell motility, posttranslational modification, protein turnover, chaperones, inorganic ion transport and metabolism, biosynthesis, transport, and catabolism of secondary metabolites, general function prediction only, cytoskeleton, were primarily upregulated in the MI group than in the HC group (*p* < 0.05). However, 10 pathways, which included defense mechanisms, translation, ribosomal structure and biogenesis, transcription, and biogenesis of cell wall, cell membrane, and envelope, were significantly downregulated in the MI group when compared to the HC group (Table 3). Overall, in the current study, the COG pathway annotation results revealed that the metabolic pathways in the patients with MI were mostly upregulated in comparison to HC.

**Table 3:** COG functional analysis between patients with MI and HC.

**Functions**	**Healthy group**	**MI group**	**Regulation in MI group**
RNA processing and modification	0.012	0.014	Up-regulated
Chromatin structure and dynamics	0.035	0.037	Up-regulated
Energy production and conversion	6.050	6.051	Up-regulated
Cell cycle control, cell division, chromosome partitioning	1.124	1.104	Down-regulated
Amino acid transport and metabolism	9.523	9.563	Up-regulated
Nucleotide transport and metabolism	3.257	3.169	Down-regulated
Carbohydrate transport and metabolism	6.758	6.840	Up-regulated
Coenzyme transport and metabolism	4.744	4.676	Down-regulated
Lipid transport and metabolism	3.364	3.340	Down-regulated
Translation, ribosomal structure, and biogenesis	7.785	7.559	Down-regulated
Transcription	6.922	6.895	Down-regulated
Replication, recombination, and repair	5.219	5.113	Down-regulated
Cell wall/membrane/envelope biogenesis	6.347	6.249	Down-regulated
Cell motility	1.418	1.453	Up-regulated
Posttranslational modification, protein turnover, chaperones	3.632	3.668	Up-regulated
Inorganic ion transport and metabolism	5.047	5.127	Up-regulated
Secondary metabolites biosynthesis, transport, and catabolism	1.625	1.659	Up-regulated
General function prediction only	11.625	11.70	Up-regulated
Function unknown	7.257	7.432	Up-regulated
Signal transduction mechanisms	4.019	4.095	Up-regulated
Intracellular trafficking, secretion, and vesicular transport	2.222	2.259	Up-regulated
Defense mechanisms	2.001	1.974	Down-regulated
Extracellular structures	0.427	0.268	Down-regulated
Cytoskeleton	0.426	0.464	Up-regulated

*The average relative abundance of COG pathway and significant difference were verified with a Wilcoxon signed-rank test in GraphPad Prism and p <0.05 suggests significant variance between both the groups*.

## Discussion

To date, the identification of bacterial species is highly dependent on culture or molecular tests (Rudkjøbing et al., 2016). The culturing of slow-growing or fastidious bacteria reveals some of the limitations of culture methods, which make identification a complicated and time-consuming process (Hasman et al., 2014). Today, development in high-throughput sequencing and improvement in targeted metagenomics workflow has facilitated their use as potential tools for quantifying and characterizing the taxonomic profile of the microbiome present in tissues, in particular blood. Several studies have reported that microbiota does exist in the blood of patients having non-communicable diseases (Amar et al., 2011b, 2013; Potgieter et al., 2015; Lelouvier et al., 2016) and even in healthy individuals (Païssé et al., 2016). Similarly, the major phyla detected in our healthy controls are consistent with those found in healthy controls of previous research studies around the world (Lelouvier et al., 2016; Païssé et al., 2016). Regarding the unavoidable contamination, we focused more on the differences in blood microbiota composition, which assists in the identification of disease-related changes. The negative control results confirmed that the microbiota in the blood is not due toreagent contamination.

To our knowledge, no comprehensive analysis of the microbiome in the blood of patients with MI has been previously performed in China. To address this gap, we performed 16S rRNA gene sequencing of microbiota DNA from the blood of MI patients and healthy individuals. Our study revealed that the peripheral blood collected from the patients and controls has diverse bacterial taxa, dominated by the phyla Proteobacteria, Firmicutes, Bacteroidetes, and Actinobacteria. This result is somewhat similar to the findings reported by previous studies. Six major taxonomic differences were observed in the blood of the MI group. At the phylum level, Actinobacteria were significantly predominant in MI patients, while Bacteroidetes were significantly abundant in HC. The increased level of association with Actinobacteria was also linked to a higher risk of cardiovascular deaths, independent of age, sex, race, dialysis vintage, and vascular access type (Sumida et al., 2021). In addition, one cross-sectional study found a significantly higher relative abundance of the Proteobacteria population in the whole blood of patients with CVD compared to that of apparently healthy individuals (Rajendhran et al., 2013). A year later, another study found that Actinobacteria were more prevalent than Proteobacteria in CVD patients, whereas the opposite tendency was seen in HC patients (Dinakaran et al., 2014; Velmurugan et al., 2020). In contrast, the increased level of Actinobacteria was observed in several other diseases, including allergic disorders such as allergic alveolitis and allergic bronchopulmonary penicilliosis (Rintala, 2011), pulmonary disease, and tuberculosis (Eshetie and Van Soolingen, 2019; Amarasekara et al., 2021). Moreover, an elevated level of Actinobacteria in diabetic nephropathy (DN) has been reported as an independent risk factor for cardiovascular mortality (Sheng et al., 2018). Similarly, our study found a significantly increased level of Actinobacteria in patients with MI (Figure 5A). However, the biological significance of the association of Actinobacteria with a specific disease is quite uncertain. In the future, shotgun metagenomic sequencing is necessary for the detailed analysis of the microbial taxa and their functions in the blood of MI patients.

Additionally, we observed that the genus level of *Bifidobacterium* increased in abundance in the MI group (Figure 5A). However, the pathogenic role of *Bifidobacterium* remains unclear. Data on the incidence of invasive infections are very limited, but *Bifidobacterium* species are estimated to represent 0.5–3% of anaerobic blood culture isolates (Cohen et al., 2016). Among adults, only 15 cases of bacteremias caused by *Bifidobacterium* had been reported in the literature until 2015 (Weber et al., 2015), and these were predominantly among patients with underlying gastrointestinal disease and/or impaired immunity. There is a paucity of data on the clinical presentations, prognostic factors, and outcomes of patients with regard to bacteremia due to infection with *Bifidobacterium*. *Bifidobacterium* species bacteremia seems to be a rare event, but its true incidence could be underestimated. Indeed, *Bifidobacterium* species could be considered as nonpathogenic bacteria, as these anaerobic, non-sporulating, Gram-positive rods are part of the physiological oral, vaginal, and intestinal flora. Besides bacteremia, urinary, pleuropulmonary, obstetric, and gynecologic infections and dental caries have been reported (Weber et al., 2015). Bacteremia due to *Bifidobacterium* species is an emerging entity, although the effects of *Bifidobacterium* in the blood of patients with MI are of importance and should be determined.

In contrast to these findings, little is known regarding the influence of alterations in the quality and composition of circulating microbiome populations in CVD. A higher (vs. lower) relative abundance of the Proteobacteria phylum in peripheral blood leukocytes was significantly associated with a higher risk of incident cardiovascular events, independent of traditional cardiovascular risk factors, in a pioneering cohort study investigating the longitudinal association between circulating microbial signatures and cardiovascular events in the general population (Amar et al., 2013). Furthermore, elevated levels of Proteobacteria in the gut and blood have also been detected in many chronic inflammatory diseases, including inflammatory bowel disease, metabolic syndrome, CVD, chronic lung diseases, and atherosclerosis plaques (Calandrini et al., 2014; Rizzatti et al., 2017). Moreover, lipopolysaccharides (LPS) have been identified as a key component produced by Proteobacteria (Amar et al., 2011b), and the role of LPS in the etiology of atherosclerosis and MI has been reported by several studies (Carnevale et al., 2020; Hashimoto et al., 2020). Hence, we conclude that Actinobacteria and Proteobacteria are the major phyla that might have a great influence on MI.

Furthermore, we measured alpha and beta diversity. Alpha diversity is used to measure the richness, evenness, and diversity of bacterial taxa within a community. In a dysbiotic gut, alpha diversity is decreased, which has been linked to autoimmune diseases, diabetes, metabolic syndrome, autism, and colorectal cancer, among other chronic diseases (Mosca et al., 2016). In addition, high vascular stiffness (Menni et al., 2018) and type 2 diabetes (Chakaroun et al., 2021), have also been associated with lower alpha diversity. Recent studies observed a lower alpha diversity in the blood of patients with chronic kidney disease (Shah et al., 2019) and MI (Amar et al., 2019). Our results were consistent with the results from previous studies, suggesting a possible association of the diseased state with lower bacterial diversity. Furthermore, we compared the taxonomy of blood bacteria between both groups. Moreover, PCoA analysis (beta diversity) of the blood bacterial composition between patients with MI and HC indicated moderate separation. A similar result was found by Kamo et al. (2017) regarding the gut microbiota in heart failure patients and HC subjects (Mayerhofer et al., 2020). The beta diversity values of the gut, oral, and thrombus microbiomes were found to be significantly different in Korean patients with ST-elevation myocardial infarction (Kwun et al., 2020). Particularly, the results of this study revealed partial separation between both groups. Taking together, this result might be an influence of beta diversity in MI.

### Functional Prediction of Blood Microbiota

Changes in diet, infectious diseases, and environmental factors alter the structure and functions of microbial composition in the host (Rinninella et al., 2019). Here, PICRUSt2 was used to estimate COG abundances using 16S rRNA gene sequencing data in patients with MI and HCs to predict functional modifications of microorganisms in the blood. The COG function pathways annotation indicated that the bacterial population in MI patients showed various functional differences (Table 3). Similar findings have been observed in atherosclerosis patients, where most of the samples were enriched by energy production and conversion, general function prediction only, and cytoskeleton (Zhou et al., 2020). The upregulation of these pathways was correlated with Huntington's disease, followed by Parkinson's disease, oxidative phosphorylation, Alzheimer's disease, and microbial metabolism in diverse environment signaling pathways (Zhou et al., 2020). Furthermore, our study found that posttranslational modifications, protein turnover, chaperones, amino acid transport, and metabolism were significantly increased in MI patients, and similar findings were reported in previous studies on different diseases (Liu et al., 2015; Wang et al., 2017; Hernández-Fernández et al., 2021), although the direct role of these pathways in MI is still not clear. According to our knowledge, the predicted functional metabolic COG pathway in MI patients was explored for the first time in our study. Regarding the future, the deep analysis of the function through transcriptomic sequence might be essential, and hence a detailed study is worth needed.

### Limitations

There are certain limitations to our study. Given the sample size limitation, larger research studies in other populations are required to validate our results on a large scale. In addition, our study is cross-sectional. Longitudinal studies of the microbiota in the blood of patients with MI at distinct periods are required. Particularly, as the blood is no longer to be recognized as “sterile,” the next target of microbiota study in CVD is atherosclerosis. To gain a better understanding of the impact of the microbiota in the pathogenesis of MI and other cardiovascular diseases, researchers must examine the microbiota in the oral cavity, blood, gut, and even skin using the 16S rRNA gene metagenome analysis or even better approaches like shotgun sequencing.

Furthermore, only blood samples were taken after the MI, making it impossible to distinguish in which section of the blood microbiome differences were identified in patients before or after the MI. This information would be extremely useful in the development of MI biomarkers and/or therapeutic targets to avoid MI and associated consequences, such as heart failure. In addition, despite its many benefits, such as high sensitivity and exhaustivity, the molecular approach based on the quantification and identification of bacterial DNA is unable to differentiate between DNA from living bacteria and DNA originating from bacterial degradation. Even if this is not a barrier to biomarker development, it makes understanding the mechanisms underlying blood microbiome diversity in individuals with MI more difficult. Moreover, other markers of intestinal leakage of inflammatory bacterial products into the circulation, such as endotoxin, could not be assessed, even though they could have aided in understanding the relationship between MI and systemic bacterial inflammation. However, our study aimed to determine that a variation of the blood microbiome exists in patients shortly after MI. From this perspective, our research offers a promising proof of concept that paves the way for the blood microbiota to be used as a diagnostic and therapeutic tool in MI. Future research should address the above-mentioned limitations.

## Conclusion

In conclusion, our study found that not only gut microbiota dysbiosis but also changes in blood microbiome composition were linked to MI. The current study revealed a lower alpha diversity and distinct variations in taxonomic profiles with a significantly high abundance of Actinobacteria phylum in the patients with MI. Previously, Actinobacteria has been linked to allergic problems, pulmonary disease, tuberculosis, and chronic kidney disease in patients. In addition, a significantly higher level of Actinobacteria reported in diabetic nephropathy (DN) is an independent risk factor for cardiovascular mortalities, demonstrating that gut bacterial translocation into the blood causes inflammation, which is a common underlying mechanism in many disorders, leading to pleomorphic modifications in the microbiological makeup of the blood. Understanding the differences in blood microbiomes will help us better understand the role of the microbiota in the pathogenesis of MI, and the possible involvement of microbiota from different tissues need to be investigated further.

## Data Availability Statement

The datasets presented in this study can be found in online repositories. The names of the repository/repositories and accession number(s) can be found in the article/Supplementary Material.

## Ethics Statement

The studies involving human participants were reviewed and approved by Medical Ethics Committee of the Northwest Minzu University, Gansu, China. The patients/participants provided their written informed consent to participate in this study.

## Author Contributions

AL conceived, initiated, and supervised the research. LZ, ZJ, and IkK designed the experiment and analyzed the resulting data. ZX and MT helped in sample collection and provided the participant's data information. YF helped in sample collection and provided sample materials. ImK and IA compiled all figures and performed the statistical analysis. MK helped in revising the manuscript. IkK wrote and ran the informatics pipeline with the help of all authors. LZ and ZJ gave conceptual advice and revised the manuscript. All authors contributed to the article and approved the submitted version.

## Funding

This study was supported by the National Natural Science Foundation of China and the project approval number is 31760159.

## Conflict of Interest

The authors declare that the research was conducted in the absence of any commercial or financial relationships that could be construed as a potential conflict of interest.

## Publisher's Note

All claims expressed in this article are solely those of the authors and do not necessarily represent those of their affiliated organizations, or those of the publisher, the editors and the reviewers. Any product that may be evaluated in this article, or claim that may be made by its manufacturer, is not guaranteed or endorsed by the publisher.
