# Supplementary material for: Comparison of Microbial Populations in the Blood of Patients With Myocardial Infarction and Healthy Individuals
Source: Front Microbiol. 2022 May 25;13:845038. doi: 10.3389/fmicb.2022.845038 (PMC9176212; doi:10.3389/fmicb.2022.845038)
Supplement: Supplementary file 1 [file Data_Sheet_1.docx]

**Supplementary data:**

**Table S1**. Categorical variables of the microbiome study. Fisher Exact test was conducted on categorical variables. The values indicate the samples numbers and their percentage.

| **Categorical variables** | **Control (n=29)** | **Cases (n=29)** | **P value**  **Fisher Exact test** |
| --- | --- | --- | --- |
| Male, n=58 | 23 (79.3%) | 23 (79.3%) | 0.999 |
| Hypertension, n=58 | 11 (37.9%) | 9 (31.03%) | 0.782 |
| Diabetes mellitus, n=58 | 6 (20.6%) | 4 (13.7%) | 0.729 |
| Active smoker, n=58 | 1 (3.4) | 15 (51.72%) | 0.0001**** |

**Table S2**. Quantitative variables of the microbiome study. Mann-Whitney tests were conducted on quantitative variables.

| **Quantitative variables** | **Healthy group**  **Median** | **25%** | **75%** | **MI group**  **Median** | **25%** | **75%** | **Mann-Whitney test** |
| --- | --- | --- | --- | --- | --- | --- | --- |
| Age, (n=58) | 54 | 47 | 68 | 56 | 50 | 67.75 | 0.414 |
| Body mass index kg/m^2^ | 24.3 | 23.4 | 26.85 | 25.4 | 23.4 | 26.05 | 0.874 |
| Systolic blood pressure mm, Hg (n=58) | 116 | 104 | 133 | 120 | 107 | 136.5 | 0.494 |
| Diastolic blood pressure mm, Hg (n=58) | 73 | 66 | 83.5 | 68 | 59 | 77.25 | 0.219 |
| TG, mmol/L (n= 58) | 1.29 | 0.98 | 1.90 | 1.13 | 0.91 | 2.01 | 0.766 |
| LDL cholesterol, mmol/L (n= 58) | 2.59 | 2.04 | 2.98 | 2.52 | 1.91 | 3.4 | 0.917 |
| HDL cholesterol, mmol/L (n= 58) | 1.16 | 1.03 | 1.31 | 1.04 | 0.85 | 1.27 | 0.086 |


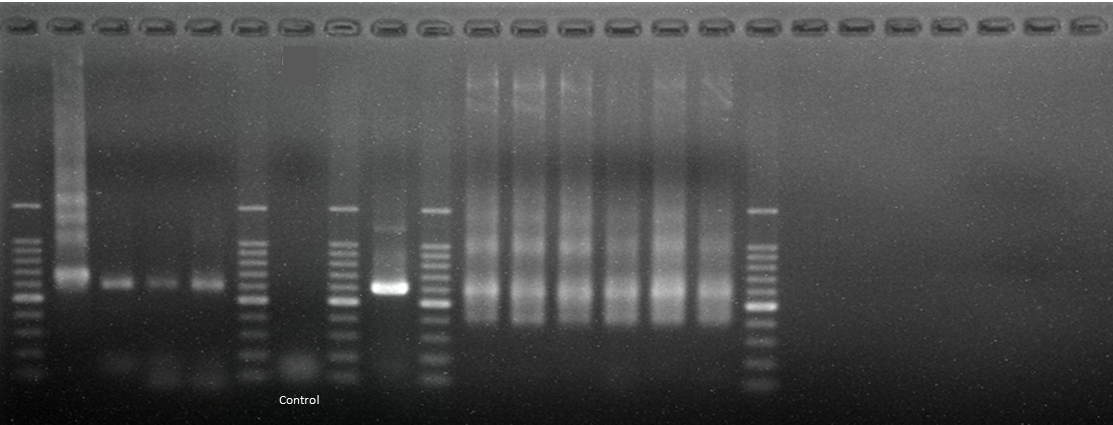


**Figure S1.** PCR results of the negative control.


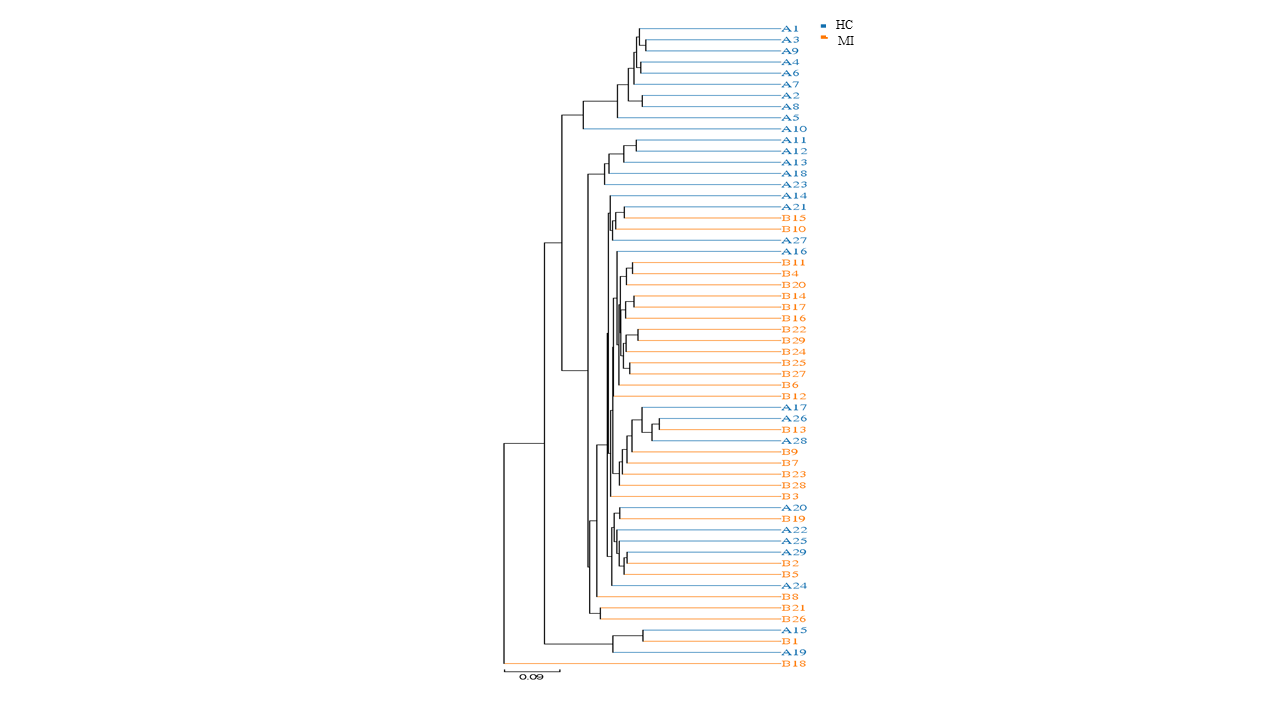


**Figure S2.** dendrogram indicates dissimilarities among all samples
